# An Effective Way of J Wave Separation Based on Multilayer NMF

**DOI:** 10.1155/2014/217067

**Published:** 2014-10-12

**Authors:** Deng-ao Li, Jing-ang Lv, Ju-min Zhao, Jin Zhang

**Affiliations:** College of Information Engineering, Taiyuan University of Technology, Taiyuan 030024, China

## Abstract

J wave is getting more and more important in the clinical diagnosis as a new index of the electrocardiogram (ECG) of ventricular bipolar, but its signal often mixed in normal ST segment, using the traditional electrocardiograph, and diagnosed by experience cannot meet the practical requirements. Therefore, a new method of multilayer nonnegative matrix factorization (NMF) in this paper is put forward, taking the hump shape J wave, for example, which can extract the original J wave signal from the ST segment and analyze the accuracy of extraction, showing the characteristics of hump shape J wave from the aspects of frequency domain, power spectrum, and spectral type, providing the basis for clinical diagnosis and increasing the reliability of the diagnosis of J wave.

## 1. Introduction

In recent years, as a new index of the ECG of ventricular bipolar, J wave draws more and more attention in the clinical diagnosis. We can consider that J wave is located between QRS wave of electrocardiogram and ST segment [1]. It represents the depolarization end and bipolarization start, under normal circumstances of the heart, ventricular finally depolarization and ventricular earliest bipolarization have the mutual transitional zone which is overlapped about 10 ms in humans, if J point deviates from the baseline, that is, the J point shift, in early repolarization syndrome, acute myocardial ischemia, pericarditis and bundle branch block, and so forth [2]. If J point is shifting and forming the special dome type or rush type, it means that J wave happened.

As the ECG signals we observed are compounded by the baseline drift, power frequency interference and myoelectricity interference noise, and so on, in most cases the original J wave signal is mixed in the normal ST segment [3]. So we cannot observe the characteristics of J wave from mixed signals. In clinic, the ECG and vectorcardiogram provide limited diagnostic information. These results are caused by some complex symptoms or clinical characteristics, and it is hard to decide the relative treatment. From the point view of signal processing, it is difficult to get accurate J wave from ECG with noise interference. It is obvious that the noise must be removed so as to get the desired information from the ECG. Then the mixed signals including J wave and normal ECG signals will be separated effectively.

It is necessary for us to study digital characteristics of J wave and we should extract J wave from ECG signal exclusively. Although the method of processing ECG signals has been very mature, methods of extracting J wave signals from ECG signal are rare to find and the extraction precision is undesirable. It will affect not only the J wave numerical characteristics directly, but also the analysis of the late classification accuracy [4]. In such situation of the clinical diagnosis, it is impossible to diagnose the healthy condition for patients with J wave. This paper puts forward a set of related methods to achieve the desired result. Commonly, the steps of signal processing can be realized as follows:

1. preprocessing the mixed signals with the method of the principal component analysis (PCA) in order to filter out noises and initialize,
2. separating the original J wave from mixed signals based on multilayer fixed-point NMF decomposition,
3. analyzing the separated J wave from point view of the spectrum, power spectrum density, and spectra in digital domain.

The simulation results show that the methods in this paper have better precision and realize the characteristics extraction of J wave. It can be used as the judgment basis for doctors.

## 2. Nonnegative Matrix Decomposition

Although the independent component analysis (ICA) and sparse component analysis (SCA) algorithm based on blind source separation (BSS) are the main methods, they are no longer applicable in practical on the condition that source signals are dependent or nonsparse. In particular, for complex ECG signal, the separation is poor. It is obvious that the nonnegative matrix factorization is a new way of blind source separation to solve the dependent source signals [5]. Therefore, this project adopts the improved NMF algorithm to extract J wave. Because the NMF has no limits of statistical independence source or sparse source, it can realize the source separation better and the algorithm has such features as fast convergence rate, sparse, nonnegative, and dimensionality reduction [6]. The optimized NMF algorithm can meet the practical requirements. The extraction results are desirable, such as extracting J wave from ECG signal accurately with small error and simple procedures. We can get more ideal extraction results.

Mathematical model of BSS problem can be expressed as follows:

(1) $$\begin{array}{c} X=AS, \end{array}$$

where *X* = [*x*_1_, *x*_2_,…, *x*_*m*_] ∈ *R* ^*m*×*N*^ is the observed mixed signal matrix; *S* = [*s*_1_, *s*_2_,…, *s*_*n*_] ∈ *R* ^*n*×*N*^ is unknown source signal matrix; and *A* ∈ *R* ^*m*×*n*^ is mixed matrix [7]. Suppose *N* ≥ *m* ≥ *n*, and *n* is known or can be estimated. The goal of BSS is to isolate unknown *A* and *S* from the mixed signal matrix *X*.

The basic model of NMF is to decompose the matrix  *V* = [*v*_1_, *v*_2_,…, *v*_*m*_] ∈ *R* ^*m*×*N*^ into the matrix of *W* = [*w*_1_, *w*_2_,…, *w*_*m*_] ∈ *R* ^*m*×*n*^ and *H* = [*h*_1_, *h*_2_,…, *h*_*n*_] ∈ *R* ^*n*×*N*^, where *W*_*ij*_ and *H*_*ij*_ are nonnegative under the limiting condition and the formula is defined as follows:

(2) $$\begin{array}{c} V=WH. \end{array}$$

Comparing (1) and (2), we can take the observation data *X* in BSS as the matrix *V* to be decomposed in NMF. If *W* and *H* are equal to *A* and *S*, respectively, then the NMF can be used to solve the problem of BSS and even the J wave separation.

## 3. The Preprocessing before Extracting

Taking the mean square error (MSE) into consideration, PCA method with the property of optimal orthogonal decomposition is the most representative in the field of signal processing and could compress ECG signal to reduce the dimension and remove the noises to get the maximum signal-to-noise ratio. It saves the subsequent decomposition time and improves the separation precision. As the final result of NMF is always the local optimum, the initialization of the objective functions *W* and *H* will affect the entire process of the NMF algorithm directly. That is to say, initializing *W* and *H* differently will produce different separation results [8]. Comparing to the traditional methods, eventually PCA is chosen for initializing. PCA can find the projection direction with the most concentrated energy in the sample space. The projection direction of the observation signal *V* will produce reconstruction signals and reconstruction error defined as $E_{\text{mse}}=E{||\overset{-}{V}-WH||}^{2}$, where $\overset{-}{V}$ is a collection standing for the mean of ECG signal; *W* is the transformation matrix; and *H* is the projection coefficient matrix. We could calculate *H* after setting a minimum range for *E*_mse_ and taking the appropriate *W*. Then we can obtain the initial transformed matrixes *W*_0_ and *H*_0_ of *W* and *H* by nonnegative processing. The procedures are *w*_*ij*_ = max⁡(0, *w*_*i*,*j*_) and *h*_*ij*_ = max⁡(0, *h*_*i*,*j*_). Therefore, PCA has great advantages of preprocessing in the NMF algorithm.

## 4. Fixed-Point NMF

Compared to the different algorithm of NMF, fixed-point NMF is suitable to extract J wave from the ECG signal. The advantage of this algorithm is that the divergence deviation is zero when *V* = *WH* [9]. We can judge the advantages and disadvantages of the algorithm by calculating the final value of corresponding objective function and the algorithm can simplify the evaluation of separation accuracy. The objective function is shown in the following formula:

(3) $$\begin{array}{c} D\left(V||WH\right)=\sum_{i,j}\left[V_{ij}\;\text{lo}{\text{g}}_{2}\frac{V_{ij}}{{\left(WH\right)}_{ij}}-V_{ij}+{\left(WH\right)}_{ij}\right], \end{array}$$

where *W* and *H* are nonnegative and when and only when *V* = *WH* is satisfied, formula (3) can obtain the minimum value 0.

The iteration rules of *W* and *H* are shown in the following formula:

(4) Hkj⟵Hkj∑iWikVij/(WH)ij∑iWik,Wik⟵Wik∑jHkjVij/(WH)ij∑vHkv,

where *W*_0_ and *H*_0_ are the initial nonnegative matrix. The divergence deviation *D*(*V*||*WH*) is monotonically increasing under the rules of iteration and the value of *D*(*V*||*WH*) is minimum and the algorithm is convergent when *W* and *H* are local optimum [10].

## 5. Selection of the Extraction Scheme

**Figure 1:**
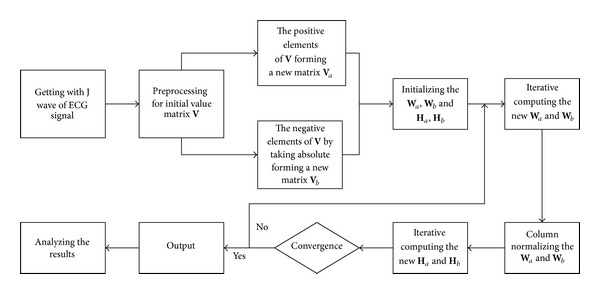
J wave extraction flowchart.

After lots of surveys, we found the constraint conditions of NMF algorithm are that the matrix elements to be decomposed are nonnegative [11]. ECG signal data acquired from a lead usually form a one-dimensional matrix. For example, we can check the specific value of ECG from Massachusetts Institute of Technology and Beth Israel Hospital (MIT-BIH) [12]. The ECG signal matrix *V* is usually composed of positive and negative elements, so the signal matrix must be improved to extract J wave from ECG by the method of NMF. In order to satisfy the limiting conditions of NMF, we classify the ECG data into positive and negative elements. The positive elements of ECG signal matrix *V* should be selected from signal matrix to form a new matrix *V*_*a*_ and the rest of the absolute value of the negative elements to form matrix *V*_*b*_. The positions of elements in the new matrices are the same as original, and other remained elements are considered as zero. *V*_*a*_ and *V*_*b*_ are decomposed, respectively, based on fixed-point NMF. The whole process can be described as *V*_*a*_ = *W*_*a*_ *H*_*a*_ and *V*_*b*_ = *W*_*b*_ *H*_*b*_. The *W*_*a*_ and $W_{\begin{array}{c} b \end{array}}$ are together a mixed matrix *W*. The *H*_*a*_ and *H*_*b*_ are together a J wave matrix *H*, where the *H*_*a*_ after decomposing *V*_*a*_ is J wave signals at the positive axis and, similarly, *H*_*b*_ is the absolute value of J wave signal at the negative axis. Therefore, the whole J wave source signal is extracted and then we can further analyze the features of hump type J wave. The J wave extraction flowchart is shown in Figure 1.

## 6. The Design of Multilayer NMF

It is difficult to get the higher separation precision with single layer NMF factorization. In particular, for some complex data and the function with local minimum, the result is not always ideal. In order to improve the performance of the NMF, this paper adopted a simple approach with hierarchy and multistage execution so that continuous nonnegative matrix factorization is performed [13].

In the first step, we use NMF algorithm to perform *V* = *W*_1_ *H*_1_ as a basic factorization. Then in second phase, we can use the same or different update rules and the results of the first stage to perform a similar factorization *H*_1_ = *W*_2_ *H*_2_. Carrying out the multilevel factorization continuously, we could get the required result at last. This process can be repeated several times until satisfying the standard. In each step, we usually make the decomposition performance gradually improve [14]. This model is *V* = *W*_1_ *W*_2_ ⋯ *W*_*L*_ *H*_*L*_ and the fundamental matrix is defined as *V* = *V*_1_ *V*_2_ ⋯ *V*_*L*_. It means that we build a system with many layers or *L* hybrid subsystem to improve the separation accuracy.

Comparing to the different layer procession of fixed-point NMF, we find that the more the number of layers is, the slower the processing speed and the extraction precision are not necessarily better. Considering the aspects of processing speed and accuracy comprehensively, we choose the 7-layer fixed-point NMF decomposition.

## 7. Experiment Results and Analysis

**Figure 2:**
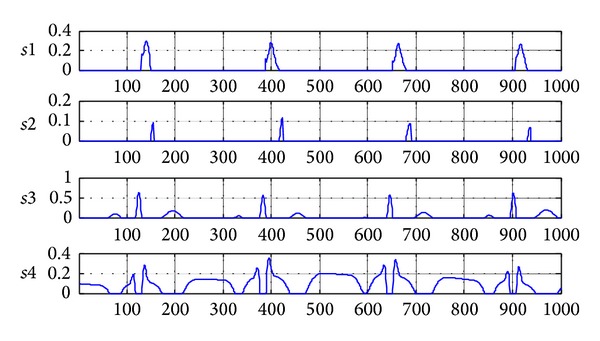
The source signals after preprocessing (*s*1 is J wave at positive axis;* s*2 is J wave at negative axis;* s*3 is normal ECG at positive axis;* s*4 is normal ECG at negative axis).

**Figure 3:**
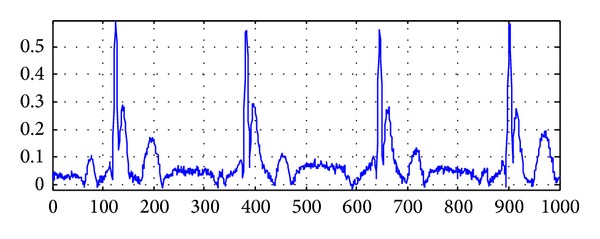
The signals after random sparse mixing.

Taking the hump J wave, for example, the figures of source signals and mixed signals are shown in Figures 2 and 3. The source signals have been preprocessed and mixed signal is generated by sparse mixed randomly. In order to make the mixed signal closer to the real signal, we need to mix the preprocessed source signals randomly. By this way, we can get a hump shape J wave that is close to the real signal and the research will be meaningful.

### 7.1. The Result of 7-Layer NMF Separation

**Figure 4:**
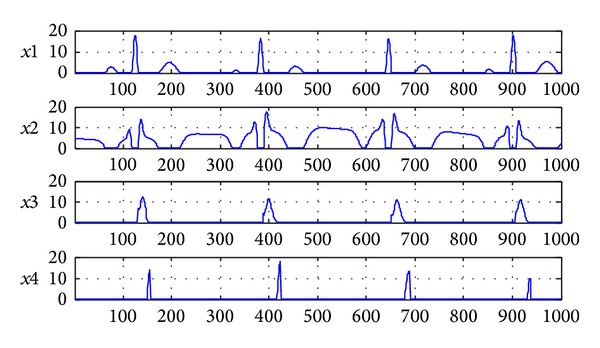
The signals after 7-layer NMF separation (*x*1 is normal ECG at positive axis;* x*2 is normal ECG at negative axis;* x*3 is J wave at positive axis;* x*4 is J wave at negative axis).

**Figure 5:**
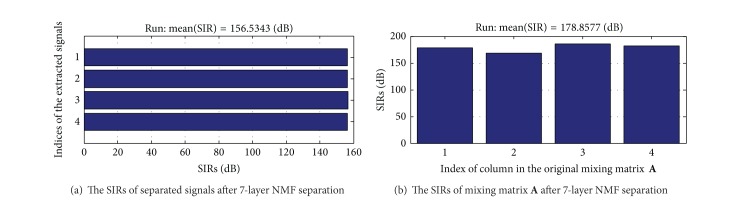
The SIRs of separated signals and mixing matrix **A** after 7-layer NMF separation.

The figures of separation results are shown in Figures 4 and 5. An estimated signal by 7-layer fixed-point NMF decomposing shown in Figure 4 is very similar to the source signals in Figure 2.  Figure 5 shows the signal interference ratios (SIRs) of estimated signal and the mixing matrix **A** after separation. We can find that the average SIR of estimated signals from four channels is 156.5343 dB and the SIR of the mixing matrix **A** is 178.8577 dB. In a word, the experiment is successful by analyzing the separation results of Figures 4 and 5.

### 7.2. The Result of Single Layer NMF Separation

**Figure 6:**
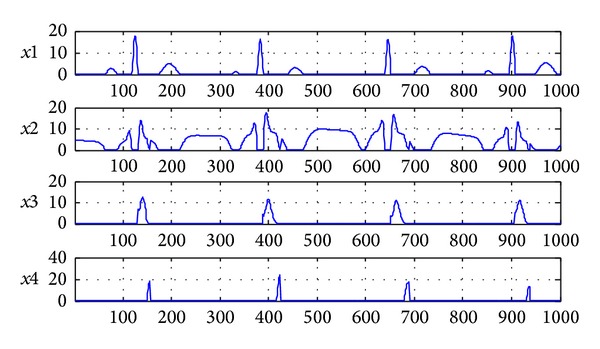
The signals after single layer NMF separation (*x*1 is normal ECG at positive axis;* x*2 is normal ECG at negative axis;* x*3 is J wave at positive axis;* x*4 is J wave at negative axis).

**Figure 7:**
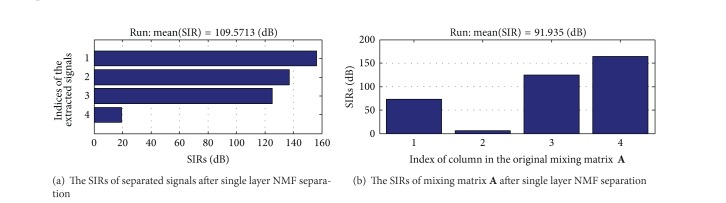
The SIRs of separated signals and mixing matrix **A** after single layer NMF separation.

As a comparison, the figures of single layer NMF separation results are shown in Figures 6 and 7.  Figure 6 shows estimated signals by single layer fixed-point NMF decomposing.  Figure 7 shows the SIRs of four-channel estimated signals and mixing matrix **A** which are 109.5713 dB and 91.935 dB. It verifies the feasibility of 7-layer separating method once again and can separate the J wave from the ECG with high accuracy. In the separated signals,* x*1 and* x*2 form a normal ECG and* x*3 and* x*4 form a hump shape J wave signal.

Compared to single layer NMF, 7-layer NMF separation is more ideal. The reason is that we cannot obtain final *W* and *H* by single layer NMF in decomposition to make the objective function converge completely, which leads to the separated signals in some areas which are different to the real signal to a great extent. The *W* and *H* obtained by one-layer decomposition of multilayer NMF can make the objective function convergent and the separated signals close to the real signals. The disadvantage is that the more the layers, the longer the time spent in the separation process. After taking comprehensive consideration, we adopt the 7-layer NMF decomposition and receive the ideal results.

### 7.3. The Result by Using ICA Separation

ICA is the most common method in BSS problem [15], of which fast independent component analysis (Fast-ICA) is the most respective. It has the advantage of fast processing speed and stabilization performance [16]. The following is the result of using Fast-ICA and the experiment result of the proposed scheme and the Fast-ICA are compared.

**Figure 8:**
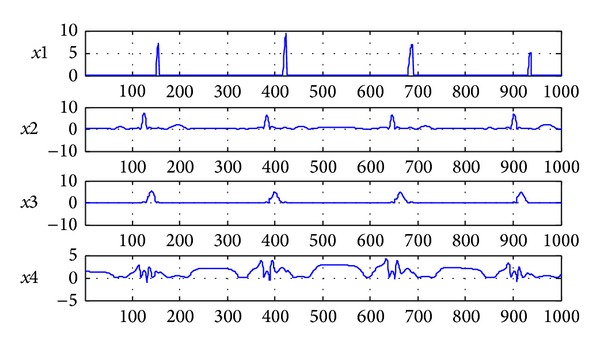
The signals after Fast-ICA separation (*x*1 is J wave at positive axis;* x*2 is normal ECG at positive axis;* x*3 is J wave at negative axis;* x*4 is normal ECG at negative axis).

**Figure 9:**
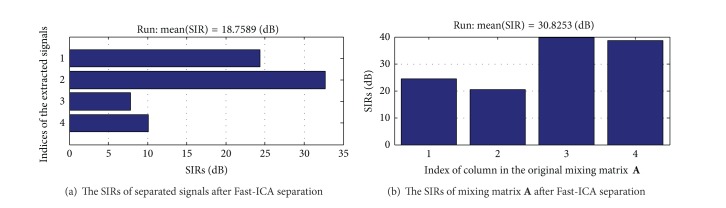
The SIRs of separated signals and mixing matrix **A** after Fast-ICA separation.

Compared to the results of our method, we can conclude that, in Figure 8, J wave and ECG signals by Fast-ICA are different from the source signals to a great extent. In Figure 9, the SIR of separating signals is 18.7589 dB, and the mixing matrix **A** is only 30.8253 dB. Through the separation effect of J wave, the proposed methods are superior to the classic Fast-ICA.

## 8. J Wave Transformation in the Field of Digital Domain

**Figure 10:**
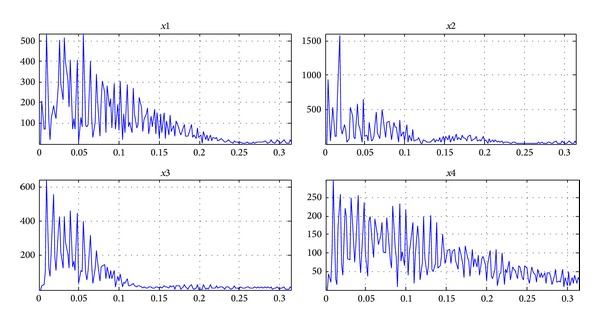
The spectrum of separated signals (*x*1 is the spectrum of normal ECG at positive axis;* x*2 is the spectrum of normal ECG at negative axis;* x*3 is the spectrum of J wave at positive axis;* x*4 is the spectrum of J wave at negative axis).

**Figure 11:**
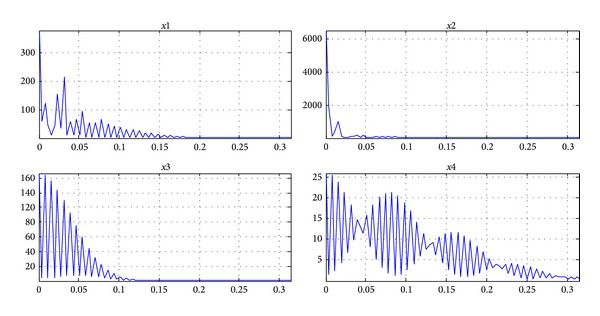
The power spectral density of separated signals (*x*1 is the spectrum of normal ECG at positive axis;* x*2 is the spectrum of normal ECG at negative axis;* x*3 is the spectrum of J wave at positive axis;* x*4 is the spectrum of J wave at negative axis).

**Figure 12:**
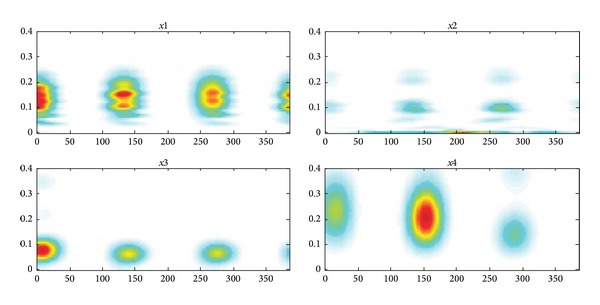
The spectrogram of separated signals (*x*1 is the spectrogram of normal ECG at positive axis;* x*2 is the spectrogram of normal ECG at negative axis;* x*3 is the spectrogram of J wave at positive axis;* x*4 is the spectrogram of J wave at negative axis).

The spectrum, power spectrum density, and spectra of the extracted hump shape J wave are shown in Figures 10, 11, and 12. Comparing to Figures 10–12, we can determine that the initial signal has the characteristics of hump-shaped J wave when they are similar to the J wave. Therefore, when the J wave is not obvious and even hidden in the ST segment, this method can be used to compare the figures of the text with the patient's ECG feature in the digital domain. Combing with the clinical experiences of doctors, they can provide a basis for doctors to judge whether the patients have an unconspicuous J wave.

## 9. Conclusion

This paper introduced a decomposing approach of multilayer fixed-point NMF and J wave can be successfully extracted from the abnormal ECG. The best initial method and decomposing layers are proposed and work well. At the same time, we conclude the spectrum, power spectrum density, and spectra of J wave in the digital domain. It can be considered as a basis for the future study of J wave characters and is helpful for doctors to diagnose the heart disease.
